# The Surgical Outcome of Anomalous Origin of the Left Coronary Artery from the Pulmonary Artery

**Published:** 2014-04-01

**Authors:** Tasneem Muzaffar, Farooq Ahmad Ganie, Sunil Gpoal Swamy, Nasir-ud-din Wani

**Affiliations:** 1 Department of Cardiovascular and Thoracic Surgery, Soura, Kashmir, India; 2 Department of Cardiovascular surgery, Pediatric Division, Amrita Institute of Medical Sciences and Research Center, Kochi, India

**Keywords:** Anomalous Origin, Mitral Valve Insufficiency, Myocardium

## Abstract

**Background:**

Anomalous origin of the left coronary artery from the pulmonary artery (ALCAPA) is a rare congenital anomaly which represents one of the most common causes of myocardial ischemia and infarction in children. This anomaly, if left untreated, results in a very high mortality rate within the first year of life. Yet, immediate surgical correction can lead to excellent results.

**Objectives:**

The present study aimed to determine the surgical outcome of ALCAPA.

**Methods:**

This study was conducted on 53 patients with ALCAPA operated from January 2005 to December 2012. Surgical repair was carried out as soon as the diagnosis was made. Surgery was thus undertaken on an urgent basis (within 48 hours) in the patients with congestive heart failure or critical clinical status and on a semi- elective basis (within a few days) in the remaining children. Operations for all the patients were performed through a median sternotomy using established standard cardiopulmonary bypass technique.

Grouped variables were compared using chi-square test with Yates’ correction. Besides, McNemar’s test was used to assess the relationship between preoperative ejection fraction and mitral incompetence. All the analyses were performed using the SPSS statistical software, version 11.5 (SPSS Inc., Chicago, IL).

**Results:**

The patients’ median age at presentation was 4 months. The mean preoperative ejection fraction was 36.5%. The results showed a significant relationship between age at presentation and impairment of ejection fraction (P < 0.001). At first, 23% of our patients presented with ejection fraction < 35%. However, 6 months after the operation, the ejection fraction improved to a mean of 53.07% (SD = 8.5) ranging from 38 - 66%. There were 5 postoperative hospital deaths with an overall mortality rate of 9.6%.

**Conclusions:**

Excellent results with desirable long-term outcomes can be achieved in the infants with ALCAPA using coronary artery implantation techniques. The best potential for recovery of the left ventricular function is in younger symptomatic infants despite the worst initial presentation. Normalization of cardiac function is expected within the first year in all operative survivors with a patent dual coronary system.

## 1. Background

Anomalous Origin of the Left Coronary Artery from the Pulmonary Artery (ALCAPA) is a rare congenital anomaly, first described by Brooks (1) in 1886. It is usually seen as an isolated lesion (2, 3) and is present in one out of 300,000 live births (0.25% to 0.5%) (3-5). It represents one of the most common causes of myocardial ischemia and infarction in children and if left untreated, results in a mortality rate of up to 90% within the first year of life (6). Surgical correction on diagnosis is the current standard (2, 7, 8). Moreover, early diagnosis and prompt surgical intervention allow excellent results. To date, the mortality rate of the disease has been reported to range from 45% to 100% (6, 9-11). Many of these deaths occurred within the early months of diagnosis due to the protocols calling for deferral of operation until the age of 1 year (12, 13). Thus, the current recommendation is surgical intervention at the time of a view, which has been supported by many who have emphasized the importance of early operation (2, 7, 8, 14-16).

Malignant ventricular arrhythmias disappear after reestablishment of a two-coronary system (17) which, if untreated, would lead to sudden death. These findings indicate the severity of the ischemic process regardless of symptomatology and justify the necessity of surgical correction as soon as the diagnosis is made, regardless of age or degree of intercoronary collateralization (18-20). Surgical correction is in fact the gold standard (21).

## 2. Objectives

The present study aims to determine the surgical outcome of ALCAPA.

## 3. Patients and Methods

This case series study was conducted in the pediatric division of Department of Cardiovascular and Thoracic Surgery, Amrita Institute of Medical Sciences and Research Center, Kochi, India.

The study was conducted on 53 patients. Surgical repair was carried out as soon as the diagnosis was made. Surgery was undertaken on an urgent basis (within 48 hours). In patients with no features of congestive heart failure and in the patients with congestive heart failure or critical clinical status on elective or semi- elective basis (within a few days) in the remaining children. Operations for all the patients were performed through a median sternotomy. Standard cardiopulmonary bypass was achieved using aortic and bicaval venous cannulation. Moderate hypothermia was used and the aorta was immediately cross clamped. Myocardial protection was instituted by either warm or cold blood cardioplegia through the aortic root in all the patients with simultaneous occlusion of the anomalous coronary artery by digital pressure or by application of a bulldog clamp to prevent run off of cardioplegia into the pulmonary circulation.

### 3.1. Statistical Analysis

Grouped variables were compared using chi-square test with Yates’ correction. Besides, McNemar’s test was used to assess the relationship between preoperative ejection fraction and mitral incompetence. All the analyses were performed using the SPSS statistical software, version 11.5 (SPSS Inc., Chicago, IL).

## 4. Results

The present study was conducted on 53 cases. The median age of the patients at presentation was four months. Additionally, 40 patients (75.5%) presented within the first 6 months and 49 ones (92.5%) presented within the first year of life. Only 4 patients (7.5%) were more than 1 year old. Besides, 29 patients (54.7%) were male, while 24 ones (45.3%) were female.

Some degree of ECG abnormality was present in all the patients. The median cardiothoracic ratio was 0.6% (range 0.57 - 0.74) and cardiothoracic ratio greater than 0.55 was observed in 85% of the patients. Moreover, the mean preoperative ejection fraction was 36.5%. The minimum ejection fraction was 16% in one patient aged about 2 months and the maximum ejection fraction was 60% in another patient who was older than 1. Ejection fraction less than 35% (severely impaired) was seen in 25 patients (47.2%) and moderately impaired ejection fraction (ejection fraction = 36 - 50%) was seen in 24 patients (45.3%). Only 4 patients (7.5 %) had a normal or mildly impaired ejection fraction (EF > 50%). Out of a total of 40 patients presenting in less than 6 months, 23 ones (57.5%) had ejection fraction < 35%. The results revealed a significant relationship between age at presentation and impairment of ejection fraction (P < 0.001).

Six months after the operation, the ejection fraction improved to a mean of 53.07% (SD = 8.5) ranging from 38 - 66%.

In addition, 23 patients who had presented with ejection fraction less than 50% preoperatively had an ejection fraction greater than 50% at the 6th month of follow-up.

In the last follow up, the mean ejection fraction was 60.89% (SD = 4.67) ranging from 49 - 67%.

Mitral valve regurgitation of varying severity was seen in 51 (96.5%) out of the 53 patients. After 6 months, however, Mitral Regurgitation (MR) was decreased to a significant level. The mean Cardio Pulmonary Bypass (CPB) time was 200.71 minutes (range: 116 - 453 minutes). Additionally, the mean aortic cross clamp time was 77.19 minutes (range: 37 - 165 minutes).

All the patients underwent aortic reimplantation of the anomalous coronary artery. Direct implantation using medial trap door technique and coronary elongation technique were performed in 44 (83%) and 9 patients (17 %), respectively. In almost all the patients, the mitral valve was not addressed at the initial surgery, even in the presence of moderate or severe MR. Furthermore, immediate sternal closure was done in 22 patients (41.5%). However, the closure was delayed to the 1st postoperative day in 24 patients (45.3%) and to the second postoperative day in 7 ones (13.2%).

There were 5 postoperative hospital deaths with an overall mortality rate of 9.6%. Among the 5 patients who died, 4 were below 6 months old and only one was older than 6 months. Congestive heart failure was the presenting symptom in 4 out of the 5 patients who died. Although the number of deaths was greater in the patients who presented with congestive heart failure, this difference was not statistically significant (P = 0.350).

### 4.1. Preoperative Ejection Fraction

In the current study, 4 (12.5%) out of the 5 patients who died had a preoperative ejection fraction of less than 35%. Although a higher mortality rate was observed among the patients with ejection fraction < 35%, the difference was not statistically significant (P = 0.640). Out of the 5 patients who died, 3 ones (9.09%) had moderate to severe MR preoperatively.

### 4.2. Follow-up

The mean duration of follow-up was 65.82 months and 3 patients did not return for follow up.

## 5. Discussion

In our series, the patients' age at presentation ranged from one month to 14 years and their median age was 4 months. Besides, 49 patients (92.5 %) presented in less than 1 year of age. In the series by B Al Soufi, the patients' median age was 5.7 months ranging from 46 days to 5.5 years (22). In the present study, 29 patients (54.7%) were male and 23 (42.3%) were female. In the series by Sanjay Theodore et al., male to female ratio was 2.5:1. In the study by Ando M et al., 4 patients were male and 9 were female (23). In Fernando Amarel's series also, 8 patients were female (73%) and 3 were male (24). Additionally, R. Neirotti et al. included 4 males and 8 females in their series (25). Furthermore, Isomatsu conducted his study on 8 males and 21 females (26). As can be seen, no definite sex predilection was observed in the investigated series.

In the present study, congestive cardiac failure was detected in 52.8 % of the patients. In the study conducted by Isomatsu also, 52% of the patients presented with congestive heart failure (26). Moreover, in the study by Fernando Amarel, 11 patients were symptomatic (27). The high incidence of heart failure as the presenting symptom in Amarel’s series was due to the fact that 8 patients presented between the 1st and the 3rd month of life. This probably reflects the end of morphological spectrum of inadequate coronary collateralization, the physiological spectrum of more severe ventricular ischemia or dysfunction, or a combination of both. In our study, out of the 40 patients presenting at less than 6 months, 89.35% presented with congestive heart failure. In Kevin Turley's series of 11 patients also, 7 patients presented with features of congestive heart failure (28). Similarly, Anthony Azaki reported that 80% of their patients presented with congestive heart failure (29).

Some degree of ECG abnormality was seen in all the present study patients. Mary Jane Barth reported Q waves in anterolateral leads in all the patients (30). Also, Guido Michelon demonstrated that Q waves in leads I and aVL were present in 22.6% of the patients (31). In Isomatsu’s study, Q waves in leads I, aVL, or both were observed in all the infants but in only 15 out of the twenty older children (26).

### 5.1. Ejection Fraction

In our series, the mean ejection fraction before surgery was 36.5%. Besides, ejection fraction < 35% (severely impaired) was detected in 47.2% of the patients. Additionally, moderately impaired ejection fraction (EF = 35 - 50%) was seen in 45.3% of the patients. Only 7.5% of the subjects had a normal or mildly impaired ejection fraction. Mary Jane Barth reported a mean preoperative ejection fraction of 18% (30). The low mean ejection fraction reported by Mary Jane Barth might be due to the fact that the patients presented in a lower age group compared to our series (30).

In the current series, MR was present in all the patients with different severities preoperatively. Yet, the degree of MR improved without mitral valve repair. T. Ojala et al. also suggested that mitral valve should not be interfered at the initial operation because it can be very difficult to repair and increases the operative risk (30).

### 5.2. Coronary Implantation Technique

In the present study, direct aortic implantation and elongation technique were used in 83% and 17% of the patients, respectively. In the study by T Ojala, direct aortic implantation was used in 69% of the patients, while coronary elongation technique was employed in 17% (32).

### 5.3. Mortality

In general, the mortality rate after dual coronary repair of ALCAPA has been reported to range from 0% to 16%. In this study, the hospital mortality rate for ALCAPA was 9.4%. Vouhe and colleagues found that preoperative left ventricular dysfunction was an incremental risk factor for postoperative mortality. This was also reflected in the results obtained by Lambert and colleagues who reported that mortality was related to lower preoperative ejection fraction (13). W. Ben Ali also conducted a study and reported the rate of hospital deaths to be 9.7%. The causes of deaths in that study were ventricular arrhythmias, neurological damage, multiorgan failure, and intractable low cardiac output syndrome (33).

In another study, Isomatsu came to the mortality rate of 6.9%. Those patients had deteriorating LV function and severe mitral incompetence preoperatively (26). Moreover, Sanjay Theodore reported a hospital mortality rate of 28.5%. In that study, the patients died due to severe myocardial failure and malignant ventricular arrhythmias (23).

### 5.4. Conclusion

A high index of suspicion and appropriate diagnostic modalities should allow rapid diagnosis of ALCAPA in any patient presenting with congestive cardiac failure. An aggressive early surgical approach is warranted as it represents the only possibility to salvage hibernating but viable myocardium. Treatment of ALCAPA aims to halt the process of myocardial ischemia and to restore the normal anatomy of the coronary arteries. Reimplantation of anomalous coronary artery to the aorta, with or without elongation techniques, must be the procedure of choice. Growing experience with the arterial switch operation and the introduction of technical modifications have made aortic relocation technically feasible even in the most unfavorable anatomic scenarios. We suggest that mitral valve repair is warranted in the selected cases. Considering the experience gained in our series, simultaneous repair of the mitral valve at the time of initial operation is probably not necessary as a general rule. Although severe MR is a risk factor for death in some series, the added ischemia time for this procedure adds to the risk in infants with very compromised ventricular function.
